# Accuracy of full-arch digitalization for partially edentulous jaws — a laboratory study on basis of coordinate-based data analysis

**DOI:** 10.1007/s00784-021-04335-3

**Published:** 2022-01-04

**Authors:** Panagiotis Kontis, Jan-Frederik Güth, Christine Keul

**Affiliations:** 1 Department of Prosthetic Dentistry, University Hospital, LMU Munich, Munich, Germany; 2 Department of Prosthetic Dentistry, Center for Dentistry and Oral Medicine (Carolinum), Goethe-University Frankfurt Am Main, Frankfurt, Germany; 3 Department of Prosthetic Dentistry, University Hospital, LMU Munich, Goethestrasse 70, 80336 Munich, Germany

**Keywords:** Accuracy, Coordinate-based data analysis, Digital dentistry, Intraoral scanner, Precision, Trueness, Digital full-arch impression

## Abstract

**Objectives:**

To compare the accuracy (trueness and precision) of direct digitization of four different dental gap situation with two IOS (intraoral scanner).

**Materials and methods:**

Four partially edentulous polyurethane mandible models were used: (1) A (46, 45, 44 missing), (2) B (45, 44, 34, 35 missing), (3) C (42, 41, 31, 32 missing), and (4) D (full dentition). On each model, the same reference object was fixed between the second molars of both quadrants. A dataset (REF) of the reference object was generated by a coordinate measuring machine. Each model situation was scanned by (1) OMN (Cerec AC Omnicam) and (2) PRI (Cerec Primescan AC) (*n* = 30). Datasets of all 8 test groups (*N* = 240) were analyzed using inspection software to determine the linear aberrations in the X-, Y-, Z-axes and angular deviations. Mann–Whitney *U* and two-sample Kolmogorov–Smirnov tests were used to detect differences for trueness and precision.

**Results:**

PRI revealed higher trueness and precision in most of the measured parameters (${\overrightarrow{V}}_{E}$ 120.95 to 175.01 μm, $\overrightarrow{V}_{E}$(*x*) − 58.50 to − 9.40 μm, $\overrightarrow{V}_{E}$ (*z*) − 70.35 to 63.50 μm), while OMN showed higher trueness for $\overrightarrow{V}_{E}$ (*y*) regardless of model situation (− 104.90 to 34.55 μm). Model D revealed the highest trueness and precision in most of the measured parameters regardless of IOS ($\overrightarrow{V}_{E}$ 120.95 to 195.74 μm, $\overrightarrow{V}_{E}$ (*x*) − 9.40 to 66.75 μm,$\overrightarrow{V}_{E}$ (*y*) − 14.55 to 51.50 μm, $\overrightarrow{V}_{E}$ (*z*) 63.50 to 120.75 μm).

**Conclusions:**

PRI demonstrated higher accuracy in the X- and Z-axes, while OMN depicted higher trueness in the Y-axis. For PRI, Model A revealed the highest distortion, while for OMN, Model B produced the largest aberrations in most parameters.

Clinical relevance

Current results suggest that both investigated IOS are sufficiently accurate for the manufacturing of tooth-borne restorations and orthodontic appliances. However, both hardware specifications of IOS and the presence of edentulous gaps in the dental model have an influence on the accuracy of the virtual model dataset.

## Introduction

Amongst others, the accuracy of indirect prosthetic restorations is determined by the accuracy of reproduction of the clinical situation. Even though conventional impression techniques have been successfully applied in dentistry for the past century, digital impression technologies dominate the modern era of patient rehabilitation, with the intraoral scanners (IOS) in the forefront [1].

Examinations of the performance of different IOS however vary significantly in their outcomes, possibly due to discrepancies in software versions, calibration, operator experience, study design, and evaluation method [2, 3]. Intraoral scanning devices utilize optical measuring principles to digitize the oral anatomy. In essence, many single images are captured by an intraoral camera and consequently stitched together with the use of a software algorithm to generate a digital model. Image overlap is however prone to errors inherent to the iteration process, which accumulate as the number of superimposed images increases, causing the overall error in the final data. This superimposition error affects the accuracy of the digital dataset and has been theorized to be dependent on several factors including the iteration algorithm, optical technology, size, and number of captured images, scanning path, distinctiveness of the captured surface, and operator experience [2].

Predominantly two different methods have been described for the assessment of the accuracy of digital models, namely, the calculation of surface differences after dataset superimposition and the metrical analysis and comparison of reference geometries [4]. Limitations of dataset superimposition with best-fit algorithms have been widely discussed, including error underestimation arising from the alignment of datasets in a most optimal position and inaccuracies generated by the iterative algorithm [5, 6]. Nonetheless, a highly accurate dataset of the clinical situation required for the calculation of reference geometries is usually difficult to obtain under in vivo conditions. In the available literature, reference geometries are mostly employed for the examination of either fully dentate arches (spheres, metal bars) or completely edentulous situations (scan bodies) [4, 7–10].

Recent studies have concluded direct digitization with IOS of single teeth, quadrants, and hemi-arches to be equivalent to or even more accurate than conventional techniques [4, 5, 11–13], while differing opinions and data exist on the accuracy of complete arch scans [14–18]. To date, little is known about the influence of edentulous areas (gaps) on the accuracy of intraoral scanning.

Several authors report lower accuracy when edentulous arches are directly digitized and have concluded optical impressions of edentulous areas to be more challenging due to the lack of distinctive anatomical features and mucosal mobility [19–21]. Yet very few studies have investigated the performance of IOS on partially edentulous dentitions [22, 23]. Therefore, the current in vitro study attempts to compare the trueness and precision of the direct digitization of four different dentitions with two IOS. The null hypotheses were that according to accuracy, there will be (H0_1) no quantitative differences between the two IOS and (H0_2) no differences between the different model situations representing different patterns of missing teeth.

## Materials and methods

### Testing models

**Fig. 1:**
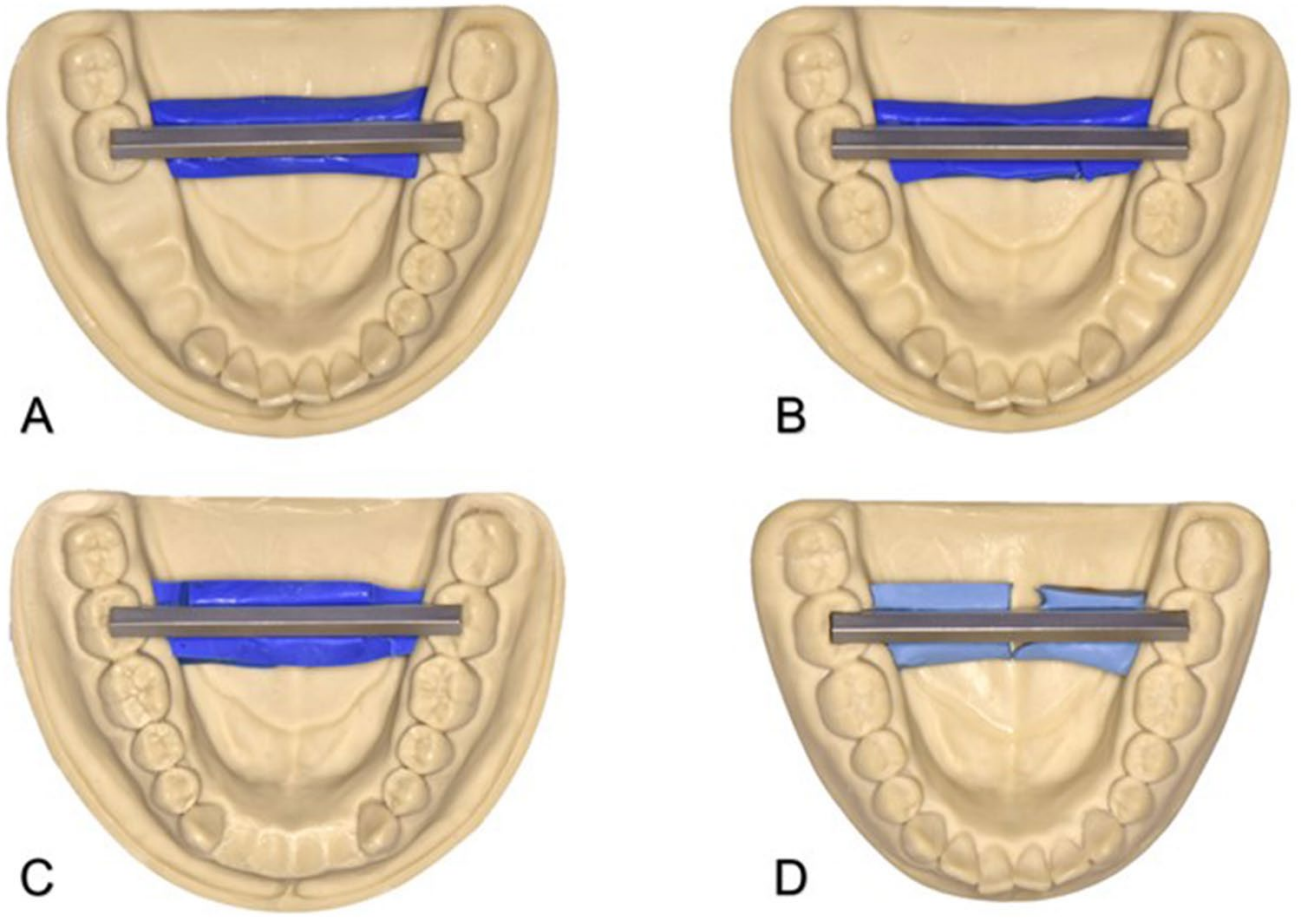
Polyurethane models with metal bar. Model A with missing teeth 46, 45, 44. Model B with missing teeth 45, 44, 34, 35. Model C with missing teeth 42, 41, 31, 32. Model D fully dentate

Four polyurethane mandible models (AlphaDie MF, LOT 2,012,008,441; Schütz Dental GmbH, Rosbach, Germany), each displaying a different partially dentate situation, were used as testing models (Fig. 1):

- Model A with missing teeth 46, 45, 44
- Model B with missing teeth 45, 44, 34, 35
- Model C with missing teeth 42, 41, 31, 32
- Model D fully dentate as control group

**Fig. 2:**
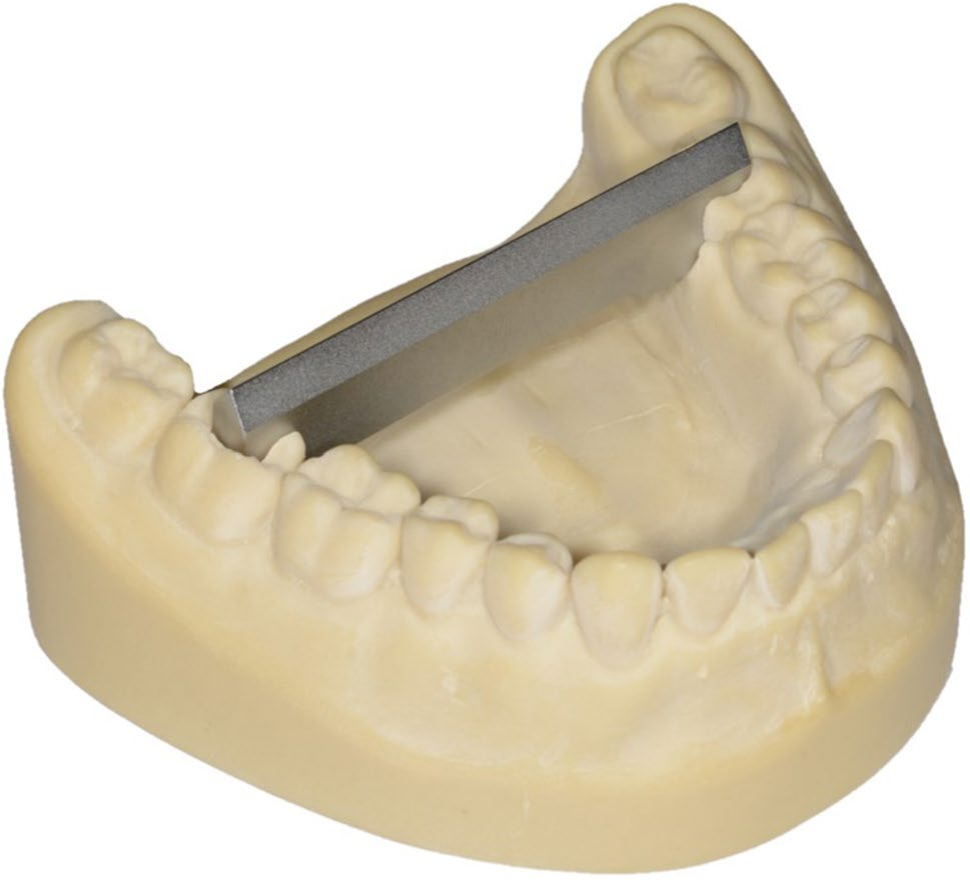
Matt surface of the metal bar on the fully dentate model

The same straight metal reference bar, made of stainless steel (GARANT, DIN 875–00-g; Hoffmann Group, Munich, Germany), was fixed between teeth 47 and 37 in each model. The surface of the bar was matt as a result of the manufacturing process (Fig. 2).

### Reference measurement and dataset of the bar

To determine the reference values of the metal bar, a measurement was performed using a coordinate measuring machine (CMM: Mitutoyo Crysta Apex C754; Createch Medical Mendaro, Spain; software: MCOSMOS Mitutoyo Software; Mitutoyo, Neuss, Germany) at a temperature of 20 °C before placing it in the model. The machine uses a 0.5 mm spherical ruby probe to measure the x-, y-, and z-coordinates of surface points on the bar. The maximum permissible error (MPEe) of the CMM is 1.9 microns + (3*L/1000) where *L* is the real bar length [24] and is calculated using the following formula: MPEe = [*k* + (multiplier * *L*)/1000] μm (*k* is the systemic or inherent length-independent error of the machine; multiplier is a constant that defines the travel dependent error, and *L* is the length of travel in millimeters).

The generated surface tessellation language (STL) dataset was imported into the inspection software (Geomagic Control 2015; Version: 2015.1.0.1919, Geomagic, Morrisville, MC, US) and analyzed analogously to the method described below for the test datasets. The reference length of the bar (*R*) and was measured to be *R* = 50.4452 mm.

### Scanning of the testing models

The four polyurethane mandible models including the reference bar were digitized with two intraoral scanners (*n* = 30/group).

- Cerec AC Omnicam (Group OMN; Software Version 5.1.1.203366, Densply Sirona, Bensheim, Germany)
- Cerec Primescan AC (Group PRI; Software Version 5.1.1.203366, Densply Sirona, Bensheim, Germany)

Both IOS were calibrated prior to each scanning session and after each five scans. All scans were performed according to manufacturer’s specifications, by the same experienced operator in the same location under ambient room lighting conditions, using the extraoral data acquisition mode. The same scanning strategy was employed for every scan, starting at tooth 48 and moving along the occlusal surface to tooth 38 then proceeding along the lingual surface back to tooth 48. Scanning concluded with the capture of the vestibular side of the dentition from the fourth to the third quadrant. During each scan, it was ensured that the opposing ends of the metal bar were not connected in the generated virtual dataset. The resulting STL datasets were post-processed and directly exported from the devices.

### Analysis of datasets

**Fig. 3:**
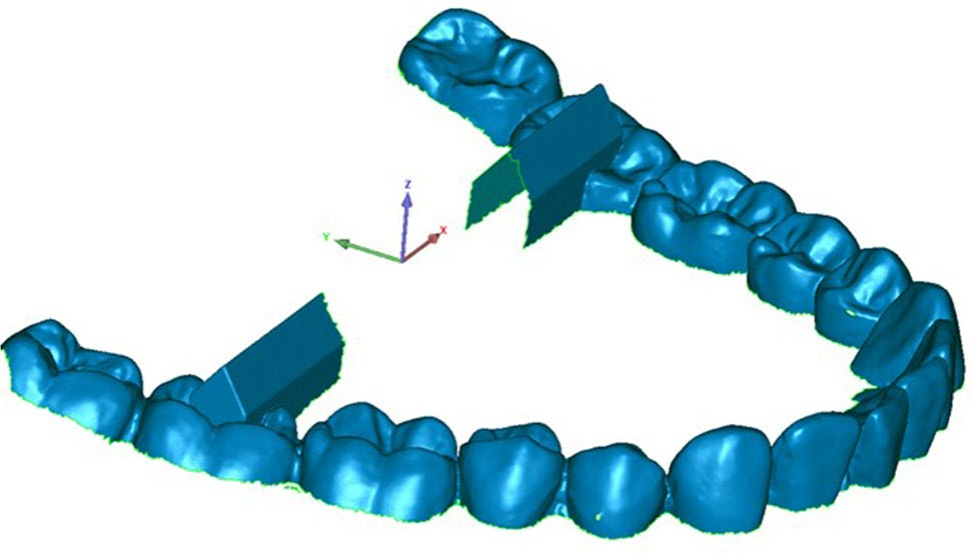
Trimmed dataset introduced in coordinate system

**Fig. 4:**
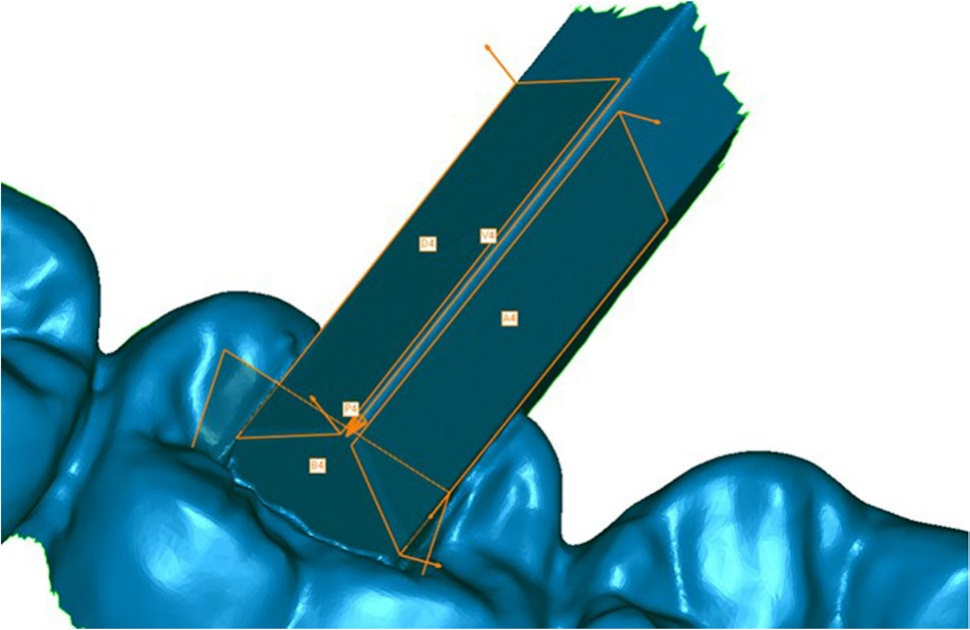
Planes A4, D4, B4, vector $\overrightarrow{\mathrm{V}}$ 4, and point P4 in the fourth quadrant

**Fig. 5:**
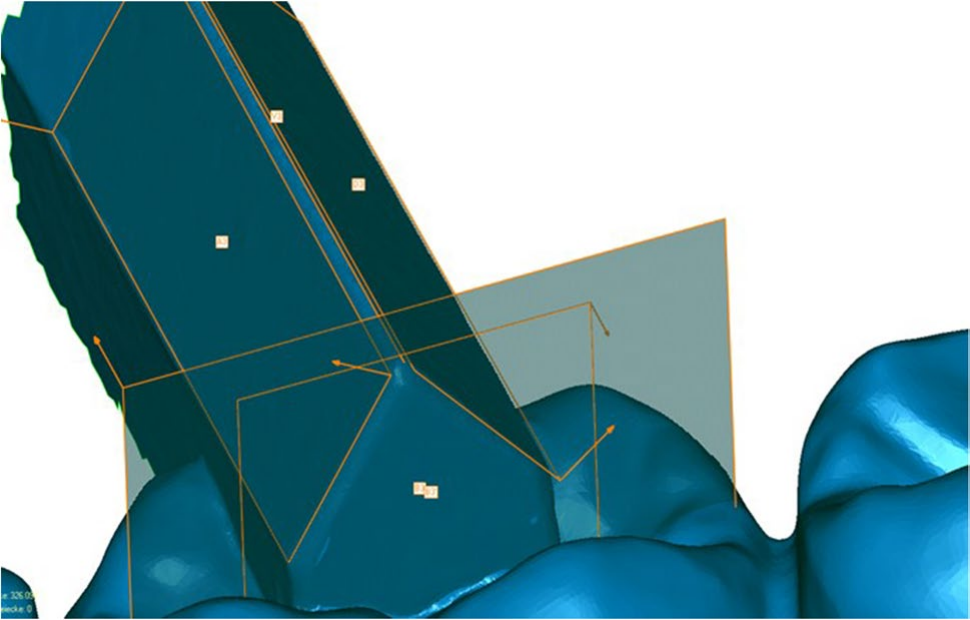
Planes A3, D3, B3, vector $\overrightarrow{\mathrm{V}}$ 3, and point P3 in the third quadrant

All datasets of both analysis groups were trimmed and equally oriented into the virtual coordinate system of Geomagic Control software, where the XY-, XZ-, and YZ-planes represent the coronal, transverse, and sagittal dimensions respectively (Fig. 3). Afterwards, the following features were generated using the software function “Contact Feature”:

- Fourth quadrant (Fig. 4): anterior plane fourth quadrant (A4), posterior plane fourth quadrant (D4), vestibular plane fourth quadrant (B4)
- Third quadrant (Fig. 5): anterior plane third quadrant (A3), posterior plane third quadrant (D3), vestibular plane third quadrant (B3)

**Fig. 6:**
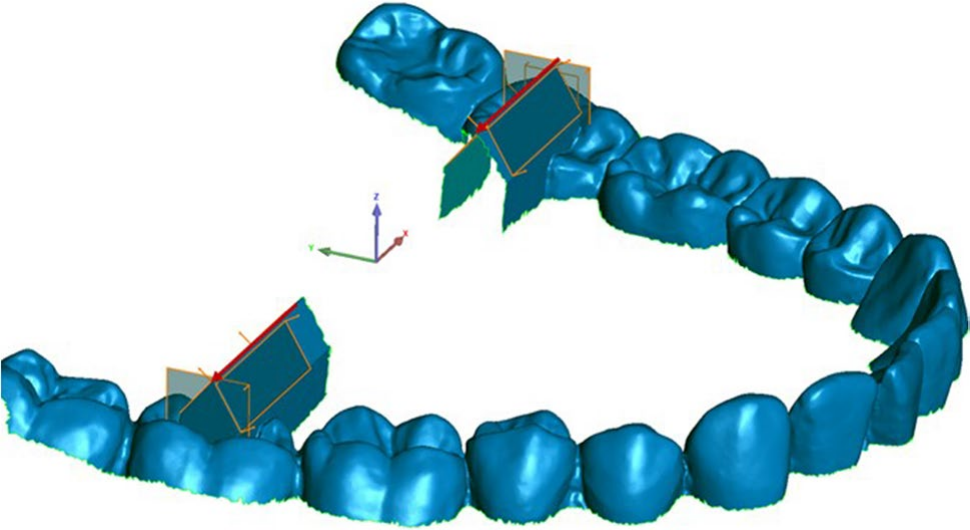
Vectors $$\overrightarrow{\mathrm{V}}$$ 3 and $$\overrightarrow{\mathrm{V}}$$ 4

**Fig. 7:**
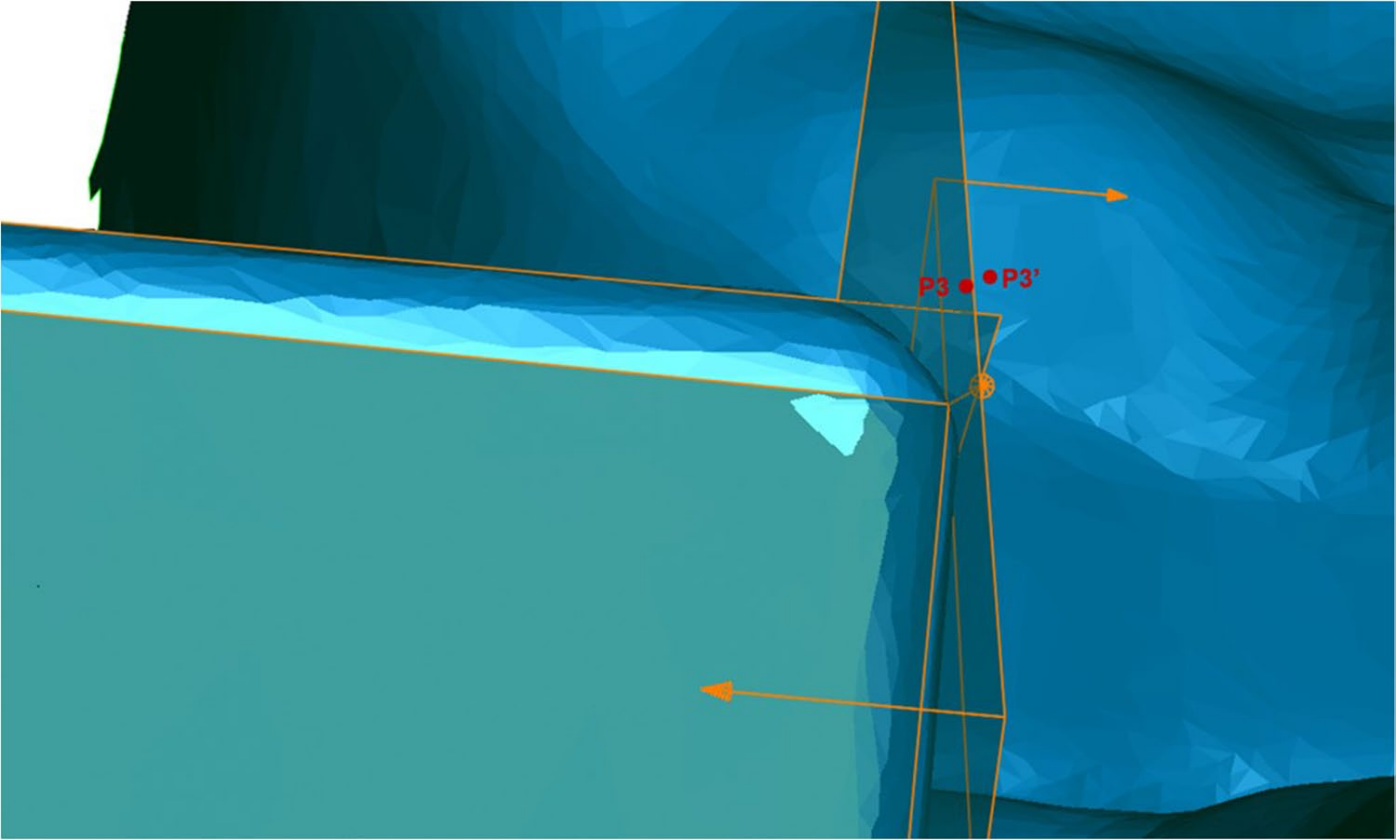
Points P3 and P3′

Vectors $\overrightarrow{\mathrm{V}}$ 3 and $\overrightarrow{\mathrm{V}}$ 4 were created at the intersection of the anterior and posterior planes in the third and fourth quadrant respectively (Fig. 6). Furthermore, the points P3 and P4 were defined as the crossing points of $\overrightarrow{\mathrm{V}}$ 3 with B3 and $\overrightarrow{\mathrm{V}}$ 4 with B4. In addition, the plane B4 was parallel shifted by 50.4452 mm in the direction of the third quadrant creating B3′ and the resulting meeting point of B3′ with the vector $\overrightarrow{\mathrm{V}}$ 3 was named Point P3′ (Fig. 7).

The point coordinates of P3, P4, and P3′ and vector coordinates of $\overrightarrow{\mathrm{V}}$ 3 and $\overrightarrow{\mathrm{V}}$ 4 were imported into Microsoft Excel (Version 1902, Microsoft Corporation, Redmond, U.S.). For the evaluation of the linear shift in the x-, y-, and z-axes, the vectoral error ($\overrightarrow{V}$_E_) was calculated between point P3′ and P3 using the following formula (x, y, and z are the coordinates of the vectors in the x-, y-, and z-axes):

$${\overrightarrow{V}}_{\mathrm{E}}=\left(\begin{array}{c}x(P3)-x(P3\mathrm{^{\prime}})\\ y(P3)-y(P3\mathrm{^{\prime}})\\ z(P3)-z(P3\mathrm{^{\prime}})\end{array}\right)$$

To assess the degree of the spatial distortion between the two halves of the bar, initially, the overall angle between vectors V3 and V4 was calculated using the following formula (X, Y, and Z are the vector parameters in the x-, y-, and z-axes):

$${\alpha }_{overall}=\alpha cos\frac{X\left(V3\right) * X\left(V4\right) +Y\left(V3\right) *Y(V4)+ Z\left(V3\right) * Z(V4)}{\sqrt{{(X(V3)}^{2}+{Y(V3)}^{2}{+Z(V3)}^{2} }* \sqrt{{(X(V4)}^{2}+{Y(V4)}^{2}{+Z(V4)}^{2}}}* \frac{180}{\pi }$$

Moreover, the projection of *α*_overall_ on the XY-plane (*α*_coronal_) and the XZ-plane (*α*_horizontal_) provides further insight about the spatial distortion between the two halves of the bar in the coronal and horizontal planes. The projections were calculated using the following formulas (X, Y, and Z are the vector parameters in the x-, y-, and z-axes):

$${\alpha }_{coronal}=\alpha cos\frac{X\left(V3\right) * X\left(V4\right) +Y\left(V3\right) *Y(V4)}{\sqrt{{(X(V3)}^{2}+{Y(V3)}^{2} }* \sqrt{{(X(V4)}^{2}+{Y(V4)}^{2}}}* \frac{180}{\pi }$$

$${a}_{\mathrm{horizontal}}=\alpha cos\frac{X\left(V3\right) * X\left(V4\right) + Z\left(V3\right) * Z(V4)}{\sqrt{{(X(V3)}^{2}{+Z(V3)}^{2} }* \sqrt{{(X(V4)}^{2}{+Z(V4)}^{2}}}* \frac{180}{\pi }$$

### Statistical analysis

For statistical analysis, SPSS Version 25 (SPSS Inc., Chicago, USA) was used. Kolmogorov–Smirnov and Shapiro–Wilk tests were applied to assess the null hypothesis, followed by Kruskal–Wallis *H* test. Trueness was evaluated using a post hoc Mann–Whitney *U* test and precision was assessed with a two-sample Kolmogorov–Smirnov test. A Bonferroni correction was applied. The level of significance was set at *p* = 0.008 for the model situation and at *p* = 0.05 for the IOS.

A post hoc power analysis by two-tailed Wilcoxon–Mann–Whitney test was conducted using G*Power software package (version 3.1.9.7). The sample size of 30 and alpha level of 0.05 was applied.

## Results

**Fig. 8:**
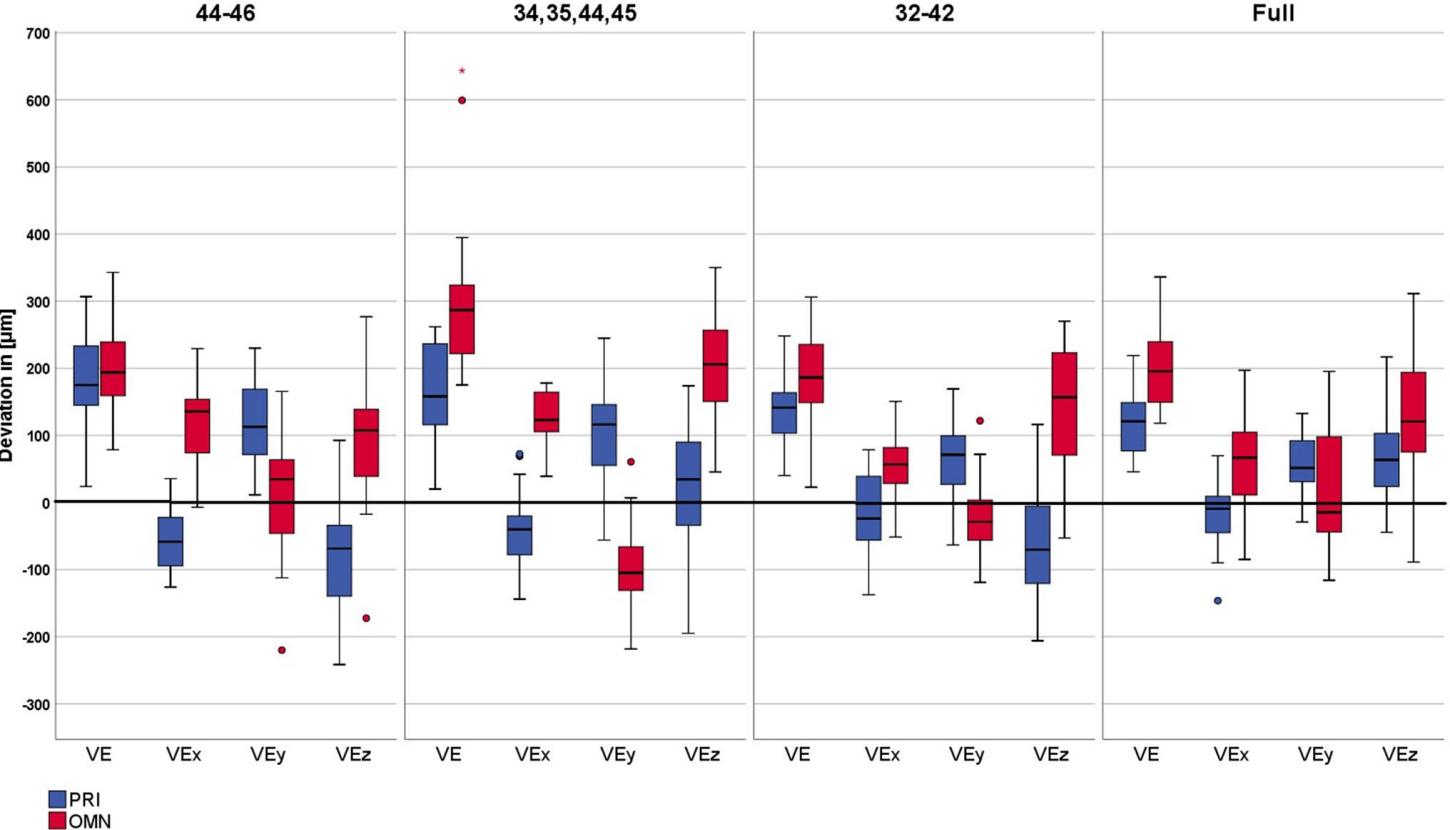
Boxplots depicting linear parameters

**Fig. 9:**
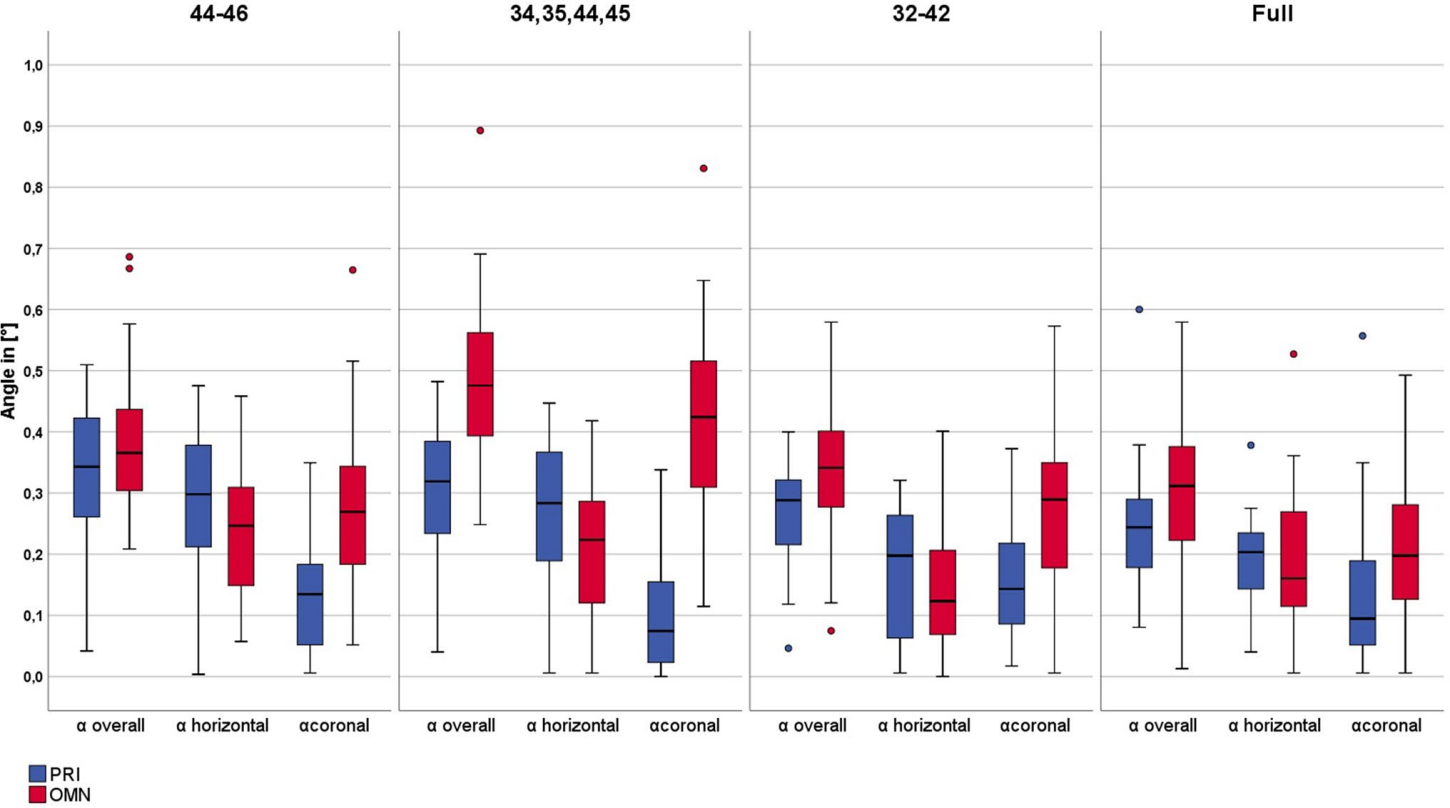
Boxplots depicting angular parameters

Descriptive statistics, including median values, minimum, maximum, and 95% confidence interval for each parameter, are given in Table 1. The Kolmogorov–Smirnov test revealed 14 out of the 56 parameters to be not normally distributed. Figures 8 and 9 show the boxplots of all tested parameters.

**Table 1:** Descriptive statistics with median values (Med), minimum (Min), maximum (Max), and 95% confidence interval (CI) and statistical analysis for all tested parameters for both IOS

Parameters		Model situation *(missing teeth)*	CEREC Primescan AC (group PRI)				CEREC AC Omnicam (group OMN)			
			Min	Med	Max	95% CI	Min	Med	Max	95% CI
Linear parameters	$\overrightarrow{V}$_*E*_ (μm)	A *(46–44)*	23.98	175.01^B/1/b/α^	306.70	151.88/205.97	78.63	*193.84^A/1/a/α^	981.77	171.67/317.55
		B *(45, 44, 34, 35)*	20.00	*158.05^B/1/b/β^	1017.87	149.22/341.95	175.24	*286.92^B/2/b/α^	643.42	256.95/336.45
		C *(42–32)*	40.17	*141.38^A,B/1/a,b/α^	893.45	105.32/214.39	22.88	186.25^A/2/a/β^	306.22	158.30/210.60
		D *(without)*	45.74	120.97^A/1/a/α^	218.91	100.03/135.89	118.03	*195.74^A/2/a/β^	336.04	172.12/214.04
	$\overrightarrow{V}$_*E*_ (*x*) (μm)	A *(46–44)*	− 126.10	− 58.50^B/1/a/α^	35.50	− 73.52/ − 37.33	− 7.10	*135.65^B/2/a/β^	229.30	95.63/136.56
		B *(45, 44, 34, 35)*	− 143.90	* − 40.10^A,B/1/a/β^	72.30	− 61.46/ − 17.22	39.10	122.95^B/2/a/α^	177.90	111.89/139.97
		C *(42–32)*	− 137.50	− 24.00^A/1/a/β^	78.60	− 33.78/9.36	− 51.50	56.75^A/2/b/α^	150.70	37.08/75.92
		D *(without)*	− 146.30	− 9.40^A,B/1/a/α^	69.80	− 37.46/ − 3.32	− 84.80	66.75^A/2/b/β^	197.00	36.54/90.02
	$\overrightarrow{V}$_*E*_ (*y*) (μm)	A *(46–44)*	11.40	112.70^C/2/b/α^	230.00	95.76/140.72	− 220.00	34.55^A/1/a/β^	165.67	− 16.99/48.43
		B *(45, 44, 34, 35)*	− 56.00	116.30^B,C/2/b/β^	244.90	74.35/130.81	− 218.20	− 104.90^B/1/b/α^	60.60	− 122.38/ − 78.38
		C *(42–32)*	− 63.30	71.25^A,B/2/a,b/β^	169.20	40.45/85.04	− 119.00	− 28.65^A/1/a/α^	121.80	− 42.13/ − 0.71
		D *(without)*	− 29.00	51.50^A/2/a/α^	132.80	40.75/72.64	− 115.90	* − 14.55^A/1/a/α^	195.40	− 17.14/46.33
	$\overrightarrow{V}$_*E*_ (*z*) (μm)	A *(46–44)*	− 241.50	− 68.55^A/1/b/α^	92.70	− 115.86/ − 50.94	− 970.90	*107.40^A/2/a/β^	276.70	− 71.17/132.28
		B *(45, 44, 34, 35)*	− 195.00	*34.40^B/1/a/β^	1016.80	− 4.68/225.15	− 631.80	*205.90^B/2/b/β^	350.10	77.43/236.27
		C *(42–32)*	− 206.00	* − 70.35^A/1/b/α^	891.50	− 104.68/35.84	− 53.00	157.00^A,B /2/a/β^	270.20	112.98/180.73
		D *(without)*	− 44.50	63.50^B/1/a/α^	216.90	48.43/94.26	− 88.70	120.75^A/2/a/β^	311.40	97.28/162.29
Angular parameters	*α*_overall_ (°)	A *(46–44)*	0.04	0.34^B/1/b/α^	0.51	0.29/0.38	0.21	0.37^A/1/a/α^	0.69	0.34/0.43
		B *(45, 44, 34, 35)*	0.04	0.32^A,B/1/a,b/β^	0.48	0.26/0.34	0.25	0.48^B/2/b/α^	0.89	0.44/0.54
		C *(42–32)*	0.05	*0.29^A,B/1/a,b/α^	0.40	0.23/0.30	0.07	0.34^A/2/a/β^	0.58	0.29/0.39
		D *(without)*	0.08	0.24^A/1/a/α^	0.60	0.21/0.29	0.01	0.31^A/2/a/β^	0.58	0.26/0.35
	*α*_coronal_ (°)	A *(46–44)*	0.01	0.13^A,B/1/a/α^	0.35	0.10/0.16	0.05	0.27^A/1/a/β^	0.66	0.22/0.32
		B *(45, 44, 34, 35)*	0.00	0.07^A/1/a/α^	0.34	0.06/0.13	0.11	0.42^B/1/b/β^	0.83	0.36/0.48
		C *(42–32)*	0.02	0.14^B/2/a/α^	0.37	0.13/0.20	0.01	0.29 ^A/1/a/β^	0.57	0.23/0.34
		D *(without)*	0.01	*0.09^A,B/1/a/α^	0.56	0.09/0.18	0.01	0.20^A/2/a/β^	0.49	0.12/0.19
	*α*_horizontal_ (°)	A *(46–44)*	0.00	0.30^B/1/c/α^	0.48	0.25/0.34	0.06	0.25^B/1/a/α^	0.46	0.21/0.29
		B *(45, 44, 34, 35)*	0.01	0.28^B/2/b,c/α^	0.45	0.22/0.31	0.01	0.22^A,B/1/a,b/α^	0.42	0.17/0.25
		C *(42–32)*	0.01	0.20^A/1/a,b/α^	0.32	0.14/0.22	0.00	0.12^A/1/b/α^	0.40	0.11/0.18
		D *(without)*	0.04	0.20^A/1/a/α^	0.38	0.16/0.22	0.01	0.16^A,B/1/a,b/α^	0.53	0.12/0.19

Not normally distributed groups are signed with *

Superscript upper-case letters indicate significant differences in trueness between different model situations, superscript numbers indicate significant differences in trueness between IOS. Superscript lower-case letters indicate significant differences in precision between different model situations, superscript Greek letters indicate significant differences in precision between IOS

The post hoc power analysis detected a power of 79 to 100% for the comparison between model situations. Regarding the comparison between intraoral scanners, power of 84 to 100% was revealed for linear parameters that demonstrated significant differences. For angular comparisons, a power of 96 to 100% was shown.

### Influence of intraoral scanner

#### Trueness

Regarding model situation A (46–44 missing), PRI performed significantly higher trueness for ${\overrightarrow{V}}_{E}$ (*x*) and ${\overrightarrow{V}}_{E}$ (*z*) (*p*<0.001) while OMN presented significantly higher trueness for ${\overrightarrow{V}}_{E}$ (*y*) (*p*<0.001). For model situation B (45, 44, 34, 35 missing), PRI showed significantly higher trueness for ${\overrightarrow{V}}_{E}$, ${\overrightarrow{V}}_{E}$ (x), ${\overrightarrow{V}}_{E}$ (*z*), *α*_overall_, and *α*_horizontal_ (*p*<0.001 to *p*=0.046), while OMN demonstrated significantly higher trueness for ${\overrightarrow{V}}_{E}$ (*y*) (*p*<0.001). Considering model situation C (42–32 missing) and model situation D (fully dentate model), PRI displayed significantly higher trueness for ${\overrightarrow{V}}_{E}$, ${\overrightarrow{V}}_{E}$ (*x*), ${\overrightarrow{V}}_{E}$ (*z*), and *α*_overall_ (*p*<0.001 to *p*=0.022) and OMN for ${\overrightarrow{V}}_{E}$ (*y*) and *α*_coronal_ (*p*<0.001 to *p*=0.022).

#### Precision

For model situation A (46–44 missing), PRI demonstrated significantly higher precision for ${\overrightarrow{V}}_{E}$ (*x*), ${\overrightarrow{V}}_{E}$ (*y*), ${\overrightarrow{V}}_{E}$ (*z*), and *α*_coronal_ (*p* < 0.001). Considering model situation B (45, 44, 34, 35 missing), OMN showed significantly higher precision for in ${\overrightarrow{V}}_{E}$, ${\overrightarrow{V}}_{E}$ (x), ${\overrightarrow{V}}_{E}$ (*z*), and *α*_overall_ (*p* < 0.001) and PRI for *α*_coronal_ (*p* < 0.001). Regarding model situation C (42–32 missing), PRI displayed significantly higher precision for ${\overrightarrow{V}}_{E}$, ${\overrightarrow{V}}_{E}$ (*z*), *α*_overall_, and *α*_coronal_ (*p* < 0.001 to *p* = 0.016) and OMN for $\overrightarrow{V}$_*E*_ (*x*) and ${\overrightarrow{V}}_{E}$ (*y*) (*p* < 0.001 to *p* = 0.003). For model situation D (fully dentate model), PRI demonstrated significantly higher precision for ${\overrightarrow{V}}_{E}$, ${\overrightarrow{V}}_{E}$ (*x*),${\overrightarrow{V}}_{E}$ (*y*), ${\overrightarrow{V}}_{E}$ (*z*), *α*_overall_, and *α*_coronal_ (*p* < 0.001 to *p* = 0.016).

### Influence of model (edentulous areas)

#### Trueness

The Kruskal–Wallis *H* test determined statistically significant differences between the different tested models for both IOS used. For PRI, model situation D (fully dentate model) presented significantly highest trueness in parameters $\overrightarrow{V}$_*E*_ (*p* = 0.001), $\overrightarrow{V}$_* E*_ (*y*) (*p* < 0.001 to *p* = 0.003), and *α*_horizontal_ (*p* < 0.001 to *p* = 0.003). Model situation B (45, 44, 34, 35 missing) exhibited significant best values for parameters $\overrightarrow{V}$_*E*_ (*z*) (*p* < 0.001) and *α*_coronal_ (*p* = 0.007). For OMN, model situations A (46–44 missing), C (42–32 missing), and D (fully dentate model) exhibited significantly highest trueness in parameters $\overrightarrow{V}$_*E*_ (*p* < 0.001), $\overrightarrow{V}$_*E*_ (*y*) (*p* < 0.001 to *p* = 0.004), $\overrightarrow{V}$_* E*_ (*z*) (*p* < 0.001 to *p* = 0.006), *α*_overall_ (*p* < 0.001 to *p* = 0.003), and *α*_coronal_ (*p* < 0.001 to *p* = 0.001). Considering *α*_horizontal_, significant best trueness was revealed by model situation C (42–32 missing) with model situations B (45, 44, 34, 35 missing) and D (fully dentate model) in the same range (*p* = 0.001).

#### Precision

For PRI, model situation D (fully dentate model) presented the highest precision in parameters $\overrightarrow{V}$_*E*_ (*p* = 0.001), $\overrightarrow{V}$_*E*_ (*y*) (*p* = 0.003), *α*_overall_ (*p* = 0.007), and *α*_horizontal_ (*p* < 0.001 to *p* = 0.001) with model situation C (42–32 missing) in the same value range. The fully dentate situation also presented significantly higher precision for $\overrightarrow{V}$_*E*_ (*z*) (*p* < 0.001) with model situation B (45, 44, 34, 35 missing) in the same value range. Considering OMN, model situations A (46–44 missing), C (42–32 missing), and D (fully dentate model) demonstrated significantly highest precision for parameters $\overrightarrow{V}$_*E*_ (*p* < 0.001 to *p* = 0.003), $\overrightarrow{V}$_*E*_ (*y*) (*p* < 0.001 to *p* = 0.003), $\overrightarrow{V}$_*E*_ (*z*) (*p* < 0.001), *α*_overall_ (*p* < 0.001 to *p* = 0.007), and *α*_coronal_ (*p* < 0.001). For $\overrightarrow{V}$_*E*_ (*x*), the significantly highest precision was shown by model situation B (45, 44, 34, 35 missing) (*p* < 0.001) with model situations A (46–44 missing) in the same value range.

## Discussion

The present in vitro study attempts to compare the trueness of two different IOS systems, namely, Cerec Primescan AC (PRI) and Cerec AC Omnicam (OMN) with four different partially edentulous situations. For that purpose, linear deviations in the x-, y-, and z-axes as well as angle measurements in the coronal and horizontal directions were examined. The investigated IOS hardware and software components used in this clinical study are currently available in the market.

In the present study, PRI presented higher trueness and precision than OMN in most of the measured parameters for every tested model. Accordingly, the first null hypothesis predicting no significant differences between the two IOS devices must be rejected. Regardless of model situation, larger discrepancies were revealed for OMN in the vertical dimension and horizontally across the arch, while PRI produced higher deviations horizontally in the anterior–posterior direction along the y-axis. Since parameters like software version, ambient light conditions, scanning strategy, calibration, and operator were maintained constant throughout the scanning procedure, the dissimilar performance of the IOS systems regarding trueness, precision, and distortion pattern can be attributed to the different hardware components or measuring principle. A significant design variation between the two devices consists in the size of the scanning head. The larger scanning unit with a bigger field of view facilitates the capture of larger parts of the arch at once, generating single images of a greater area, which can be more precisely overlapped by the software algorithm, thusly alleviating inaccuracies generated during the stitching process. A prior study reported improved accuracy on partially edentulous arches when a larger scanning head was used, while Kim et al. attributed the inability of OMN in digitizing a partially edentulous arch to the smaller scanning head of the device [25, 26]. On the other hand, a bulky handpiece might limit maneuverability in the oral cavity. Additionally, scanning of the steep interproximal surfaces of the teeth may produce distorted images and therefore generate a greater error due to improper image overlap. The occurrence of higher discrepancies in the unilateral edentulous situation with PRI could be accounted for by the substantially sized scanning head which hindered access to the gap between teeth 47 and 43. Secondly, the two systems utilize a different optical capturing technology, which is regarded to be crucial in determining the depth of image stitching and could therefore account for the difference in the vertical dimension displayed by the two IOS [8, 9, 27, 28]. OMN relies on active triangulation with a strip light projection, where accuracy error is determined by the angle of light reflection and is dependent on the distance between camera and object [28–30]. Consequently, abrupt changes of scanning depth, for instance in edentulous areas, may negatively influence the accuracy of digitization and account for the larger discrepancies in the vertical dimension demonstrated by OMN. Moreover, the lower trueness and precision in model situation 45, 44, 34, 35 illustrates the cumulative distortion caused by edentulous areas on each side of the arch. PRI works on the basis of shortwave light with optical high-frequency contrast analysis for dynamic depth scan and high-resolution sensors and seems to be less affected by changes in the focus level. However, due to the different working principles, the ambient scanning light conditions might have a different influence on the accuracy results of IOS. For the Cerec AC Omnicam, the ambient light intensity significantly influenced the trueness and precision of the virtual model dataset after direct digitalization [31]. However, for the Cerec Primescan AC, no literature information was available, so this should be a topic of further investigation.

**Fig. 10:**
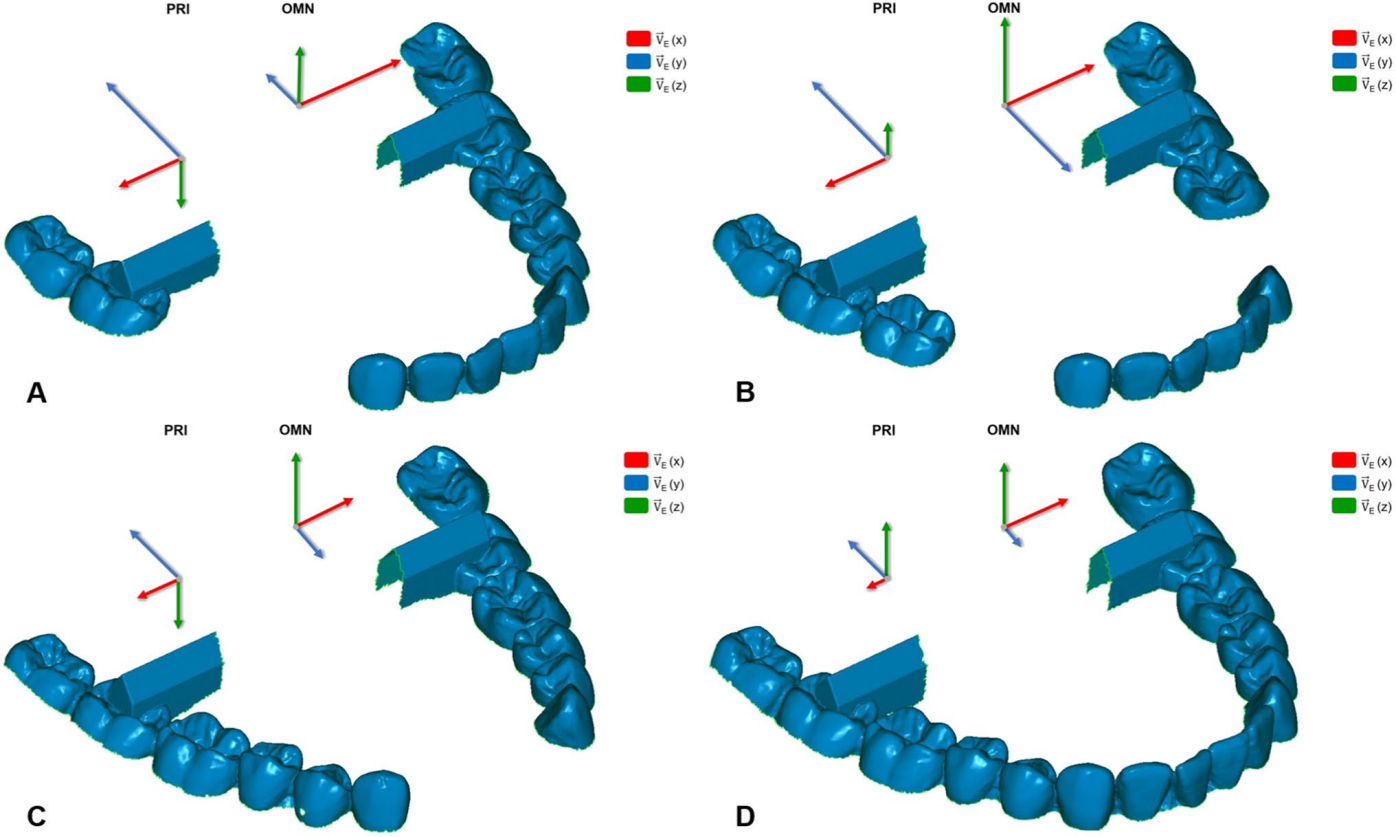
Deviation pattern produced by each IOS for every model situation. Direction and length of the pictured arrows indicate spatial drift of the dataset in length and direction for model situations A, B, C, and D

The second null hypothesis, stating that no differences in accuracy should arise between the different model situations, also must be rejected as the fully dentate model situation exhibited significantly higher trueness and precision in most tested parameters regardless of IOS. Figure 10 depicts the different patterns produced by each scanner for every model. With PRI the anterior edentulous situation displayed significantly lower trueness in $\overrightarrow{V}$_*E*_ (*z*) and *α*_coronal_ to the fully dentate situation signifying that the lack of anterior teeth produces higher vertical deformations. In addition, significant differences between the fully dentate and the bilateral partially edentulous model in $\overrightarrow{V}$_*E*_, *α*_horizontal_, and $\overrightarrow{V}$_*E*_ (*y*) indicate that edentulous areas in both halves of the dental arch increase model warpage horizontally in the anterior–posterior direction along the y-axis. Model situation missing 46–44 differed significantly in almost every parameter to the fully dentate situation, demonstrating that a larger edentulous area located closer to the origin of the scan path tends to generate overall higher error and lower predictability. By contrast, digitization with OMN seems to produce a different pattern of distortion. Model situation missing 42–32 performed similarly to the fully dentate situation. Several authors maintain that the steep and narrow surfaces of the anterior teeth amplify the error generated by image overlap, thusly increasing the overall inaccuracy of the dataset [7, 32–34]. Considering the current results, however, this theorized effect of the anterior teeth appears to have been overestimated. Moreover, model situation missing teeth 46–44 showed significant divergence to the fully dentate model in the $\overrightarrow{V}$_*E*_ (*x*) and *α*_horizontal_, possibly because of transversal expansion along the x-axis. The bilateral edentulous situation exhibited significantly higher aberrations to the fully dentate model and lowest predictability in every direction, signifying that edentulous spaces in both hemiarches result in lower overall accuracy.

The results were reported for trueness and precision according to ISO 5725 [35]. For better comparison of the results with the current literature, an established methodology was used [4, 12]. Also, the use of reference data acquired by highly accurate industrial digitalization units is the gold standard for reporting trueness. In most of the scientific literature, datasets of digital models are quantitatively analyzed after superimposition with a reference dataset employing a best-fit algorithm, while deviation patterns are usually calculated on basis of 3-dimensional difference [33, 34, 36, 37]. The calculated results are often given as positive and negative deviations from the reference dataset and graphically depicted in color-coded maps. However, this approach has been subject to criticism, primarily due to errors inherent with data processing of full arch digital models [4, 5, 18, 27]. Moreover, as best-fit alignment attempts to minimize the overall differences between datasets, real errors may be inevitably obscured [3, 6, 7]. Therefore, a qualitative comparison of the results with the available literature is challenging. Linear deviations produced by PRI in the x-, y-, and z-axes are within the range of values reported in a previous in vivo examination [18]. By contrast, a study by Kim et al. revealed in vitro higher linear discrepancies for OMN in the y- and z-axes, albeit on a partially edentulous mandible with scan bodies in the area of the missing teeth [10]. A recent in vitro study found significantly higher trueness for PRI than for OMN in the x- and y-axes, yet no significant differences could be found in the z-axis [9]. Kuhr et al. reported higher vertical angular deviations for OMN on fully dentate mandible in vivo [7]. Furthermore, PRI has been repeatedly shown to perform better than OMN, although it should be noted that the aforementioned comparisons are conducted with dataset superimposition after best-fit alignment [32, 34, 37]. Regarding warpage of virtual models, previous investigations based on color-coded maps have reported anterior contraction and posterior expansion of datasets generated with OMN [32, 36].

The findings of the current research suggest that the presence of edentulous areas in the dental arch, especially in place of posterior teeth, negatively affects the trueness of the generated digital model. Due to the absence of distinctive anatomical structures and the presence of non-attached mucosa or saliva, the digitization of edentulous spaces is rendered particularly challenging [19, 26, 38]. The inaccuracies and distortions in the digital model affect the subsequent computer-assisted design and manufacturing procedure and eventually influence the accuracy of the prosthetic restauration. Discrepancies in the transverse and horizontal planes (x- and y-axes) could translate in a misfit of the final restauration, while vertical divergence (z-axis) illustrates the torsion between the two hemiarches and might eventually result in occlusal incongruity.

Prior investigations analyzed the influence of varying edentulous anatomies on the accuracy of resulting virtual model dataset by best-fit superimposition [25, 39, 40] and corroborate the current results. However, to the author’s knowledge, no previous in vitro study has investigated the effect of varying partially edentulous anatomies on the accuracy of full-arch digital impressions using a reproducible reference structure for all investigated model situations. Though by superimposition of datasets, no quantifiable information on the generated pattern of distortion can be provided, a more detailed surface analysis can be achieved. In general, with the current setup, only the accuracy of the initial step of the workflow, namely, the digital impression, can be investigated; hence, errors bearing on the manufacturing process cannot be assessed. To analyze the accuracy of the complete workflow, including the manufacturing of the dental restorations, the final fit of the dental restoration should be investigated [41].

Advantageous of the current analyzing methodology is the applicability of the comparison between different digital impression systems [4, 5], model morphologies — even dysgnathic situations [42] — and potentially in an in vivo setting [4]. However, like every scientific work, the present work is subject to several limitations. Scanning performance was evaluated only for the lower jaw, as digitization of the upper jaws has been theorized to result in higher accuracy due to the additional image overlap over the palatal area [7]. Further on, the present study was conducted in a laboratory setting where the effect of multiple factors such as patient movement, patient compliance, spatial restrictions, and the presence of saliva or blood, which may influence the results of in in vivo experiment, cannot be reproduced [33]. However, a recent investigation by Keul et al. revealed a comparable pattern of distortion for in vivo and in vitro scans [4]. In addition, the polyurethane models exhibit different optical properties to intraoral structures (enamel, dentin, mucosa), therefore IOS may perform differently when scanning intraorally [43]. Furthermore, only one experienced operator was included, and the data acquisition was performed not in a clinic-simulating situation using a phantom head. Therefore, the data capturing mode of both IOS systems were switched to extraoral digitalization. Lastly, future scientific research is necessary to address the effect of different scanning strategies on partially edentulous situations as well as to analyze the accuracy of IOS on a greater variety of partially edentulous jaws.

The results of the current investigation suggest that Cerec Primescan AC and Cerec AC Omnicam are applicable in digital prosthetic planning, complex implant planning for fixed prosthodontics on edentulous jaws, and even digital planning of complex orthognathic procedures [42, 44]. Moreover, both systems provide sufficient accuracy for the manufacturing of tooth-borne restorations and orthodontic appliances, as the measured error falls within the range of tooth mobility [45]. However, for full-arch fixed implant restorations, where passive fit is required [46], the use of intraoral scanning systems should be considered with reservation, even though the use of IOS for the manufacture of fixed implant prosthesis based on the “All-on-4” concept with implants placed in the area between the second premolars has been documented [44].

## Conclusions

Within the limitations of the present in vitro study, the following conclusions can be drawn:

1. Cerec Primescan AC exhibited higher trueness and precision than Cerec AC Omnicam in most tested parameters regardless of scanned model situation or anatomy.
2. Considering model situation, the highest trueness and precision were demonstrated by the fully dentate model.
3. For Cerec Primescan AC, model situation A (missing teeth 46–44) was revealed to be less accurate, while for Cerec AC Omnicam, model situation B (missing teeth 45, 44, 34, 35) produced the lowest accuracy in most parameters.
4. Scanning head size and optical capturing technology seem to influence the accuracy of digitization.
