# Correction to: Accuracy of full‑arch digitalization for partially edentulous jaws — a laboratory study on basis of coordinate‑based data analysis

**DOI:** 10.1007/s00784-022-04418-9

**Published:** 2022-02-21

**Authors:** Panagiotis Kontis, Jan‑Frederik Güth, Christine Keul

**Affiliations:** 1 Department of Prosthetic Dentistry, University Hospital, LMU Munich, Munich, Germany; 2 Department of Prosthetic Dentistry, Center for Dentistry and Oral Medicine (Carolinum), Goethe-University Frankfurt Am Main, Frankfurt, Germany; 3 Department of Prosthetic Dentistry, University Hospital, LMU Munich, Goethestrasse 70, 80336 Munich, Germany

**Correction to: Clinical Oral Investigations**

**https://doi.org/10.1007/s00784-021-04335-3**

In the published paper, the author names were inverted.

The original article has been corrected.

